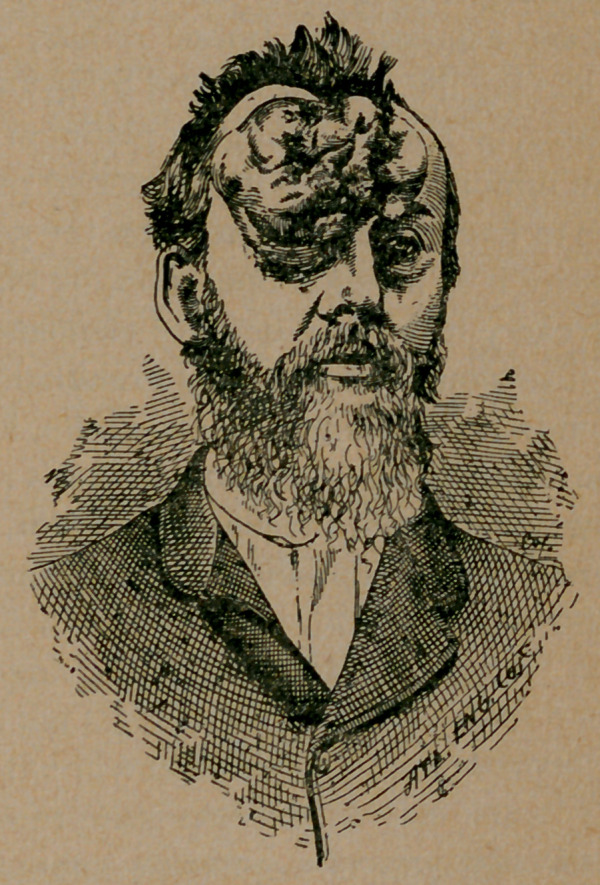# A Fibro-Sarcoma—The Advisability of Its Removal

**Published:** 1890-05

**Authors:** W. L. Bullard

**Affiliations:** Columbus, Ga.


### A FIBRO-SARCOMA—THE ADVISABILITY OF ITS REMOVAL.*

W. L. BULLARD, M. D., COLUMBUS, GA.

MR. PRESIDENT — Doubtless you are already cognizant of the fact that in the beginning of his career, the physician, surgeon or specialist is apt, as his patients present themselves, to seek, like the judge of our courts, for a precedent; but he will soon discover, whatever has been his hospital or clinical advantages, that all the ills the world is afflicted with are not in the books, and between his professional pride and sympathy his skill, judgment and nerve will at times be surely taxed. It has been my luck to fall heir to several cases not on the card, or so vague as to fail me a precedent, but none more interesting and puzzling than the one I have the honor to present to-day. This unfortunate patient is now 57 years of age. He consulted me a little over two years ago, saying that two months previous he had been stricken with severe pain over the eye, which, however, lasted only a short time. He had already consulted a physician who prescribed some eye drops. When I first saw the case, there existed no external abnormality, save an insufficiency of the internal superior rectus muscle; but on palpation I discovered a small growth or tumor situated in the upper part and nasal side of the orbital cavity behind the ball. I so informed the patient and advised an operation, which was declined. Not being allowed to operate or extirpate the growth by surgical means, I prescribed kali iodidi in increasing doses with cor. sub. Several weeks later, when patient presented himself, he also could detect the tumor, though he still objected to an operation. At this time there existed optic neuritis, and vision, which was $\frac{xx}{200}$, continued to decrease to complete blindness. The specific remedies seemed

*Read before Georgia Medical Association, Brunswick, April 18, 1890.

to have no effect, so I put him on arsenic as advised for malignant growths by Dr. Long, of Cuthbert, Ga. This treatment was tried for a time ; but the growth continued, like Banquo's ghost, to rise. By this time the tumor had bulged from the forehead, and had attained the size of a large apple, perfectly smooth, and to the touch as hard as bone, giving every indication of an exostosis, but with a needle its whole depth could be easily penetrated down to the frontal bone, which disabused our minds of the idea of having a bony tumor to contend with. Shortly after this, from its increase in size, and from constriction of the blood vessels, its natural color and smoothness changed to that of a congested and purplish hue and convoluted roughness, as you now see it. At this stage I tried electrolysis, using six to ten cells, but being quite painful was not used persistently as it should have been, for I am inclined to think that this somewhat checked its further development.

After a number of weeks, which was about seventeen months ago, I had permission to operate surgically; but at this stage, on account of its size and no evidence of its being incapsulated, I

was dubious as to what to do, but decided to cut down into the growth and see what could be done. So, with the help of my friends, Drs. Hurt, Walker and Blanchard, I etherized the patient, and with knife divided the tumor the whole length down to the bone; but, as expected, the skin was infiltrated—indeed, there was no line of demarcation between the skin and tumor; and thus being deprived of a flap, was forced to suspend the operation. The incision was washed with bichlor. sol., brought together, and held in apposition by a number of sutures, and healed by first intention. When I saw him several months afterwards, the tumor had somewhat changed, so much so that the constricted conjunctiva, as shown in the cut or photograph taken just before the operation, had all disappeared; in fact, there seems to be no undue pressure in the orbital cavity, the place where first discovered; but the eye is covered by the tumor hanging over it from above. This unfortunate man is still asking for help; hence my appeal to you and this body. I frankly admit that I am doubtful as to its favorable prognosis, yet when I remember when in London several years ago, I might, on any Thursday, visit St. Bartholomew's Hospital, and, perchance, there find such men as Messrs. Lawson, Nettleship, Bryant, Errichsen, Paget, Lister, Butlin, Heath and other eminent surgeons, with cases simulating Charcot's disease, abdominal and intra-orbital tumors, the diagnosis and prognosis of which they could not without a mental reservation positively swear to, I am not at all embarrassed by my frankness in supplicating you—yes, all of you, though particularly the experienced surgeons—of the advisability of etherizing the patient and excising the tumor *in toto*, and with antiseptic dressing, leaving the wound to heal by granulation, or like Neftel and Semmola, or Naples, plunge a platinum anode perpendicularly into the tumor, down to its presumed point of implantation, with three to five cathodes placed on the periphery of the growth, and destroy it at a single operation, by closing the current, and rapidly carry it to forty or sixty elements, charring the growth, leaving it to slough and heal by granulation.

The patient is now, and has been all the time, in good health, with no syphilitic history, or cancerous diathesis ; and as seeing is believing, I now, gentlemen, present my patient, Mr. Jno. Bennett, for your personal examination and consideration.

## Figures and Tables

**Figure f1:**
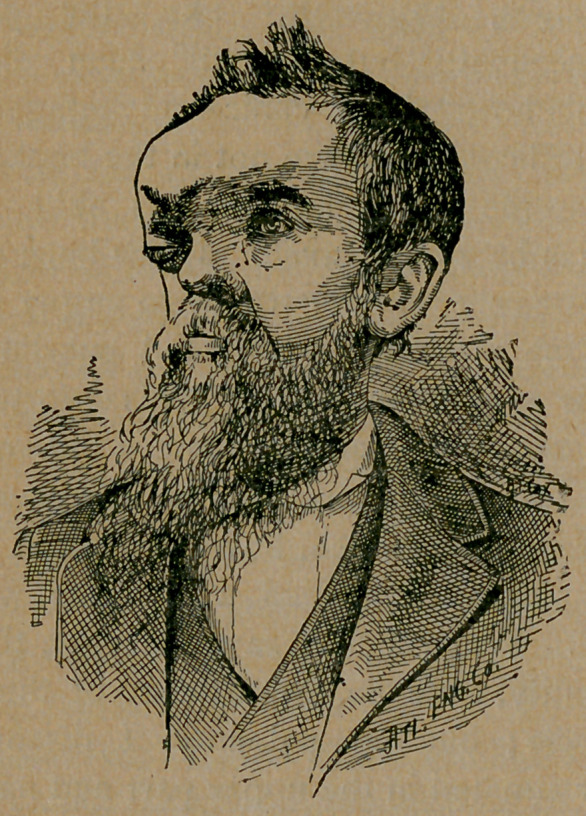


**Figure f2:**